# The Philosophy of Health; or, an Exposition of the Physical and Mental Constitution of Man, with a View to the Promotion of Human Longevity and Happiness

**Published:** 1835-04-01

**Authors:** 


*The Philosophy of Health; or, an Exposition of the Physical and Mental Constitution of Man, with a View to the Promotion of Human Longevity and Happiness.* By Southwood Smith, m.d., Physician to the London Fever Hospital, &c. *Vol. I.*—London, 1835. 12mo. pp. 408.

The rapid multiplication of hygienic works has certainly been accompanied of late by an improvement in their quality, and we have now to congratulate the public on the appearance of a volume of this kind, from the pen of so accomplished a physician as Dr. Southwood Smith. The book is written for the laity, and, though we do not mean to fall into the agreeable hypocrisy of supposing that every practitioner is perfectly acquainted with every thing contained in it, still it would be injudicious to fill our pages with elementary matter, and we must therefore content ourselves with a very brief notice of this laudable work.

Dr. Smith, we observe, from the dedication, is one of those who suppose quarantine to be "monstrously absurd." We would refer those who are willing to see the arguments on the other side admirably stated, to Dr. Gooch's paper appended to his treatise on the Diseases of Women.

Perhaps the most interesting chapter in the work is the fourth, which treats of the relation between longevity and happiness, and enters at some length into the statistics of mortality in ancient and modern times. An extract will show the tone of this part of the treatise.

"Hitherto, in all places which man has made his abode, noxious agents have been present which act injuriously upon his body, tending to disturb the actions of its economy, and ultimately to extinguish life. All these noxious agents, of whatever name or quality, may be included under the term Causes of Mortality. Inherent in the constitution of the body are conservative powers, the tendency of which is to resist the influence of these causes of mortality. The actual mortality at all times will of course be according to the relative strength of these destructive agents, and the relative weakness of these conservative powers. There are states of the system tending to enfeeble these conservative powers. Such states become tests, often exceedingly delicate, of the presence and power of the destructive agents to which the body is exposed; and such, more especially, are, the states of parturition, infancy, and sickness. During the prevalence of these states, in which the conservative powers of the body are weak, life is destroyed by causes which do not prove mortal in other conditions of the system. Accordingly, in every age and country, the rate of mortality among its lying-in women, its infants and its sick, may be taken as a measure of the degree in which the state of the whole population is favourable or unfavourable to life.

"The change that has taken place in the condition of lying-in women during the last century in all the nations of Europe cannot be contemplated without astonishment. The mortality of lying-in women in France, at the Hôtel Dieu of Paris, in 1780, is stated to have been one in 15. In 1817, for the whole kingdom of Prussia, including all ranks, it was one in 112. In England, in the year 1750, at the British Lying-in Hospital of London, it was one in 42; in 1780, it diminished to one in 60; in the years between 1789 and 1798, it further decreased to one in 288; in 1822, at the Lying-in Hospital of Dublin, it was no more than one in 223; while during the last fifteen years at Lewes, a healthy provincial town, out of 2410 cases there have been only two deaths, that is, one in 1205. There is no reason to suppose that the mortality in the state of parturition is less at Lewes than in any other equally healthy country-town in England.

"Equally striking is the proof of the diminished violence of the prevalent causes of disease and death derived from the diminished mortality of children, the vital power of resistance being always comparatively weak in the human infant, and consequently the agents that prove destructive to life exerting their main force on the newborn, and on those of tender age. From mortuary tables, preserved with considerable accuracy at Geneva since the year 1566, it appears that, at the time of the Reformation, one half of the children born died within the sixth year; in the seventeenth century, not till within the twelfth year; in the eighteenth century, not until the twenty-seventh year: consequently, in the space of about three centuries, the probability that a child born in Geneva would arrive at maturity has increased fivefold. In the present day, at Ostend, only half of the newborn children attain the age of thirty; whereas, in England, they attain the age of forty-five.

"No less remarkable is the progressive diminution of mortality among the sick of all ages. Hippocrates has left a statement, which has come down to our times, of the history and fate of forty-two cases of acute disease. Out of this number, thirty-seven were cases of continued fever; of these thirty-seven febrile cases, twenty-one died, above half of the whole. The remaining five were cases of local inflammation, and of these four were fatal: thus, of the whole number of the sick (forty-two), twenty-five were lost. Now, even in the Fever Hospital of London, to which, for the most part, only the worst cases that occur in the metropolis are sent,—and even of these many not until so late a period of the disease, that all hope of recovery is extinct,—the mortality rages in different years from one in six to one in twelve; and, for a period of ten consecutive years, it is no more than one in seven; while, in the Dublin Fever Hospital, where most of the cases are sent very early, the average mortality, from 1804 to 1812, was one in twelve." (P. 138.)

We trust that our author will not take us for croakers, if we doubt the strength of the position which he has taken up at the beginning of this quotation. He asserts that, "in every age and country, the rate of mortality among its lying-in women, its infants, and its sick, may be taken as a measure of the degree in which the state of the whole population is favourable or unfavourable to life." Of course, nobody would deny the proposition, if by *sick* the sick of the whole community were meant; but our author evidently means the sick in hospitals. How does the case stand then? At St. Petersburg the mortality in the Imperial Hospital is one in four and a half, at Göttingen it is one in twenty-one. (P. 141-2.) Surely the chances of life do not differ in the same proportion in these two towns. Nor does the progressive diminution of mortality in the same hospital show an equally great diminution in the national mortality; for it is obvious that the directors of hospitals are more accessible to knowledge than the mass of the community. St. Thomas's and St. George's are scoured and whitewashed, while St. Giles's laughs scavengers and cholera committees to scorn.

We must also take the liberty of correcting an error at the end of the quotation. It is the identical mistake of Sir G. Blane's, which we set right on a former occasion, (*Med. Quart. Rev.*, vol. iii., p. 150.) He supposed that forty-two cases of acute disease, narrated by Hippocrates, of which twenty-seven terminated fatally, might be taken as a fair average of his practice, and would therefore show the great superiority of ours. But what reason Sir Gilbert or our author could have for supposing these to be average cases, does not appear. Dr. Abercrombie, in his masterly works on the Diseases of the Brain, and of the Abdominal Viscera, has detailed an immense majority of fatal cases; yet it would be unreasonable to deduce as a conclusion, that one of the first physicians of the age loses nineteen out of twenty patients. The plain fact is, that he who possesses the "fiduciam magnarum rerum" often selects the most instructive, i. e. the most unsuccessful cases for comment; and this whether he is of Cos or of Edinburgh.

One more extract, and we must have done: it is from the chapter on the Circulation.

"That the arterial tubes do possess and exert a truly vital power, modifying the current of the blood they contain, is indubitably established.

"1. If in a living animal the trunk of an artery be laid bare, the mere exposure of it to the atmospheric air causes it to contract to such a degree, that its size becomes obviously and strikingly diminished. This can result only from the exertion of a vital property, for no dead tube is capable in such a manner of diminishing its diameter.

"2. If during life an artery be opened and the animal be largely bled, the arteries become progressively smaller and smaller as the quantity of blood in the body diminishes. If the bleeding be continued until the animal dies, and the arteries of the system be immediately examined, they are found to be reduced to a very small size; if again examined some time after death, they are found to have become larger, and they go on growing successively larger and larger until they regain nearly their original magnitude, which they retain until they are decomposed by putrefaction. ·

"3. M. Poiseuille distended with water the artery of an animal just killed. This water was urged by the pressure of a given column of mercury. The force of the re-action of the artery was now measured by the height of a column of mercury which the water expelled from the artery could support. It was found that the artery re-acted with a force greater than that employed to distend it, and greater than the same artery could exert some time after death; but since mechanical re-action can never be greater than the force previously exerted upon it (293), it follows that the excess of the re-action indicated in this case was vital.

"4. If an artery be exposed and a mechanical or chemical stimulus be applied to it, its diameter is altered, sometimes becoming larger and sometimes smaller, according to the kind of agent employed.

"299. Any one of these facts, taken by itself, affords a demonstration that the arterial trunks and branches are capable of enlarging and diminishing their diameter by virtue of a vital endowment. There is complete evidence that the exertion of this vital power on the part of the arterial trunk is not to communicate to the blood the smallest impulsive force; the engine constructed for the express purpose of working the current generates all the force that is required; but the labour of the engine is economized by imparting to the tubes that receive the stream a vital property, by which they wholly remove the physical obstructions to its motion." (P. 394.)

